# Superparamagnetic Nanoparticles with Efficient Near-Infrared Photothermal Effect at the Second Biological Window

**DOI:** 10.3390/molecules25225315

**Published:** 2020-11-14

**Authors:** Maria Antònia Busquets, Juan Marcos Fernández-Pradas, Pedro Serra, Joan Estelrich

**Affiliations:** 1 Department of Pharmacy, Pharmaceutical Technology and Physical Chemistry, Universitat de Barcelona, Avda Joan XXIII, 27-31, 08028 Barcelona, Catalonia, Spain; mabusquetsvinas@ub.edu; 2 Institut de Nanociència i Nanotecnologia (IN^2^UB), Universitat de Barcelona, 08028 Barcelona, Catalonia, Spain; jmfernandez@ub.edu (J.M.F.-P.); pserra@ub.edu (P.S.); 3 Department of Applied Physics, Universitat de Barcelona, Martí i Franquès 1, 08028 Barcelona, Catalonia, Spain

**Keywords:** NIR, superparamagnetic nanoparticles, photothermal therapy, photothermal agents, biological windows

## Abstract

Superparamagnetic nanoparticles (iron oxide nanoparticles—IONs) are suitable for hyperthermia after irradiating with radiofrequency radiation. Concerning the suitability for laser ablation, IONs present a low molar absorption coefficient in the near-infrared region close to 800 nm. For this reason, they are combined with other photothermal agents into a hybrid composite. Here, we show that IONs absorb and convert into heat the infrared radiation characteristic of the so-called second-biological window (1000–1350 nm) and, in consequence, they can be used for thermal ablation in such wavelengths. To the known excellent water solubility, colloidal stability and biocompatibility exhibited by IONs, an outstanding photothermal performance must be added. For instance, a temperature increase of 36 °C was obtained after irradiating at 8.7 W cm^−2^ for 10 min a suspension of IONs at iron concentration of 255 mg L^−1^. The photothermal conversion efficiency was ~72%. Furthermore, IONs showed high thermogenic stability during the whole process of heating/cooling. To sum up, while the use of IONs in the first bio-window (700–950 nm) presents some concerns, they appear to be good photothermal agents in the second biological window.

## 1. Introduction

Thermal therapy encompasses all therapeutic treatments based on conduction of heat into or out of a part of or the whole body [1]. The physiologic repercussion of this transfer of thermal energy is getting a temperature that is either colder or hotter than normal. Concerning the medical applications of hot temperatures, thermal therapy may be accomplished by two techniques, namely, hyperthermia—including long-term low-temperature hyperthermia with treatment for 6–72 h at 40–41 °C and moderate temperature hyperthermia with treatment for 15–60 min at 42–45 °C—and thermal ablation or high-temperature hyperthermia, with exposure to >50 °C for 4–6 min [2]. Hyperthermia treatments are an approach of great interest, especially in oncology. Low and moderate-temperature hyperthermia results in changes in the physiology of the tissues that are for the most part reversible. For this reason, such treatment must be combined with other therapeutic approaches like radiation or chemotherapy to be a valid anti-cancer treatment. In contrast, thermal ablation produces more dramatic and irreversible changes as vascular stasis, protein denaturation, cellular coagulation and necrosis [3].

Thermal ablation requires the transformation of energy into heat. To this end, many different energy sources can be used: Radiofrequency, high-intensity focused ultrasonography, microwave, alternating magnetic field and laser [4]. Concerning the use of a laser, a photothermal interaction is produced by the transformation of photon energy (absorbed by tissue fluids) into heat energy that emanates from the molecular vibration and collisions between molecules. A portion of the vibrational energy of excited molecules is transferred to the colliding molecule as kinetic energy of translation. This transmission is manifested on the macroscopic scale as a temperature increase [5]. However, thermal ablation using a visible laser has been normally regarded as a non-reliable technique mainly for three reasons. First, the existence of the so-called heat-sink effect that depletes heat and weakens the power of the thermal effect. This effect hinders the treatment of lesions near large vascular structures using laser ablation alone [6]. Secondly, human tissues strongly absorb the radiation of the visible range of the electromagnetic spectrum; this fact limits any photothermal treatment to superficial tumors [7,8]. Furthermore, as both healthy and cancerous tissues can absorb the energy of a visible laser, damage in non-cancerous tissues is also possible. To solve these problems and increase the efficacy and selectivity of photothermal ablation induced by laser, it is imperative to use radiation at wavelengths with low absorption in tissues and include light-absorbing materials, the photothermal agents (PAs), in the tumor. Thus, controlling the incorporation of PA into tumors facilitates a high heat deposition in the tumor area at low laser intensities and thus minimizes the injury in the surrounding tissue [9]. Moreover, further diminution of non-desired light absorption by healthy tissues can be reached by using specific laser wavelengths belonging to the so-called biological windows [10].

Biological windows can be considered as the spectral ranges in which tissues become to a certain extent transparent due to a simultaneous reduction in both absorption and scattering. Within these windows, three singular wavelength regions have been established: The so-called first biological window that extends from 700 nm to 950 nm. This window limits with the visible band and an absorption band at 980 nm due to the absorption of water. Skin, tissues and hemoglobin display minimal absorbance at this range. The second biological window covers the region from 1000 nm to 1350 nm, both limits corresponding to water absorption bands. In this spectral region, optical absorption does not disappear thoroughly but, on the other hand, scattering is reduced because of the longer wavelengths. The third biological window is found from 1350 to 1870 nm and provides increased transparency toward biological matter [11]. The wavelengths of these biological windows belong to the near-infrared (NIR) range of the electromagnetic spectrum.

The simultaneous use of PA and lasers emitting radiation at wavelengths in the range of biological windows is the basis of the photothermal therapy (PTT). Unlike photodynamic therapy (PDT) which has an anticancer effect relying on the generation of radical oxygen species (ROS), PTT exerts its effect by increasing the temperature of the milieu [12]. In photothermal ablation induced by laser, the cancer treatment provided does relatively little damage to surrounding healthy tissues because the thermal effect is solely produced when NIR light is applied and only in the presence of a PA which transforms absorbed light into heat. An ideal PA should possess large absorption cross sections for optical wavelengths, have low toxicity and be soluble in biocompatible solutions [13].

Organic and inorganic materials can act as PA [14]. Among inorganic materials, some nanostructures are suitable as theranostic systems. For instance, magnetic nanostructures can display the dual functions of NIR absorption and magnetism. The magnetic property affords the capacity of being used as a magnetic resonance imaging (MRI) contrast agent, and the use of an external magnetic force facilitates the enrichment of the magnetic nanostructures in the desired local tumor regions, while they enable the conversion of NIR irradiation into heat for PTT.

Among magnetic nanoparticles with dual functions of NIR absorption and magnetism, iron oxide nanoparticles (IONs) attract attention as particles suitable for PTT. IONs can be prepared in different sizes and shapes by means of well-established and relatively cheap methodologies. IONs are biodegradable, biocompatible, relatively non-toxic and may be easily functionalized and tuned for specific applications. Iron oxide can be present in different chemical compositions, such as magnetite (Fe_3_O_4_), maghemite (γ-Fe_2_O_3_) or, most usually, a non-stoichiometric mixture of the two. Nanosized magnetite is known to naturally oxidize, at least partly, toward maghemite, thus getting to an unknown composition of the tested product [15]. Below specific sizes (25 nm for magnetite, 30 nm for maghemite), both oxides present superparamagnetism behavior; that is, in the presence of an external magnet superparamagnetic nanoparticles turn to magnetic, but return to a non-magnetic state when the external magnet is removed [16]. IONs can be easily degraded in vivo, where iron homeostasis is assumed by the iron-storage protein, ferritin [17,18]. Furthermore, IONs are excreted mainly by feces, thus indicating low damnification for organisms [19].

The crystalline structure of magnetite consists of a cubic inverse spinel. The face-centered cubic (fcc) unit cell (JCPDS 19–629) is composed of 32 O anions, 16 Fe(III) cations and 8 Fe(II) cations. Half of the Fe(III) cations are tetrahedrally (tet) coordinated, while the other half and all of the Fe(II) ions are octahedrally (oct) coordinated, forming a unit cell of (Fe_8_^3+^)_tet_(Fe_8_^3+^ F_e8_^2+^)_oct_O_32_ [20]. Fe(II) and Fe(III) in the octahedral sites result in an intervalence charge transfer (IVCT) that rises to an absorption band at the second NIR region at 1000–1350 nm [21]. When magnetite becomes maghemite by oxidation, Fe^2+^_oct_ is oxidized to Fe^3+^_oct_, producing vacancies restricted to the octahedral sites. In order to explain these vacancies, the unit cell for maghemite (JCPDS 39-1346) can be expressed as (Fe_8_^3+^)_tet_(Fe_40/3_^3+^ □_8/3_)_oct_O_32_ where □ indicates a vacant site. Finally, it is important to remark that an outer coating of maghemite may develop at the particle–water interface during magnetite oxidation [22]. In this case, the non-stoichiometric structure of maghemite results in a loss of optical absorption in the NIR wavelengths regions [21].

IONs proved to be suitable for hyperthermia when they were irradiated with radiofrequency of 224 MHz using a waveguide applicator [23]. Concerning the suitability for PTT, IONs possesses a low molar absorption coefficient in the NIR region, and thus an apparent poor photothermal efficacy. For this motive, usually they are associated with other PA forming a hybrid nanocomposite, especially with gold or with an organic compound [14]. However, IONs can be effective PAs themselves. It was demonstrated that the greatest part of the heat generated by magnetite nanoparticles was effectively dissipated to the surroundings without producing significant undesirable changes of the particles such as crystalline phase transitions, agglomeration or fragmentation [24]. Recently, the photothermal effectiveness of IONs has been proved to depend on particle size: The effectiveness shows a good correlation with particle volume [25]. Several works using aggregated or individual IONs as PA in the first biological window have been described [26,27,28,29,30,31,32]. In this way, Yuan et al. demonstrated that IONs coated with polyethylene glycol (PEG) presented anti-cancer activity [33]. However, to the best of our knowledge, there are only few studies on the photothermal therapy application of IONs in the second biological window [34,35].

The aim of the work here described was to show the photothermal properties of IONs illuminated with the radiation of the second biological window. There is growing interest in the second NIR window, which offers better light penetration, lower background signal and higher maximum permission exposure compared to the traditional first window [36]. Concretely, the NIR first window is not optimal because the auto-fluorescence of the tissues results in an important background noise, and the presence of photon scattering restricts the tissue penetration depth. It is known that photon scattering scales as *λ^−α^*, where *λ* is the wavelength and *α* = 0.2–4 for biological tissues. In consequence, scattering is lesser if a radiation with longer wavelengths is used. The maximum permissible exposure (MPE) for skin is 1 W/cm^2^ for a 1064 nm laser, compared with 0.3 W/cm^2^ for a radiation of 808 nm, according to the ANSI (American National Standard Institute) standard (ANSI Z 136.1 and Z 136.3 combination set: “Safe Use of Lasers and Safe Use of Lasers in Health Care Facilities”). The same Institute indicates that the exposure time can reach 3 × 10^4^ s (~1 day).

## 2. Results and Discussion

### 2.1. Characterization of IONs

IONs were synthesized by coprecipitation of iron salts in the presence of PEG. The obtained ferrofluid was characterized by X-ray diffraction (XRD), transmission electron microscopy (TEM), high-resolution TEM (HRTEM), dynamic light scattering (DLS), Doppler microelectrophoresis, thermogravimetric analysis (TGA) and content in iron.

**Figure 1:**
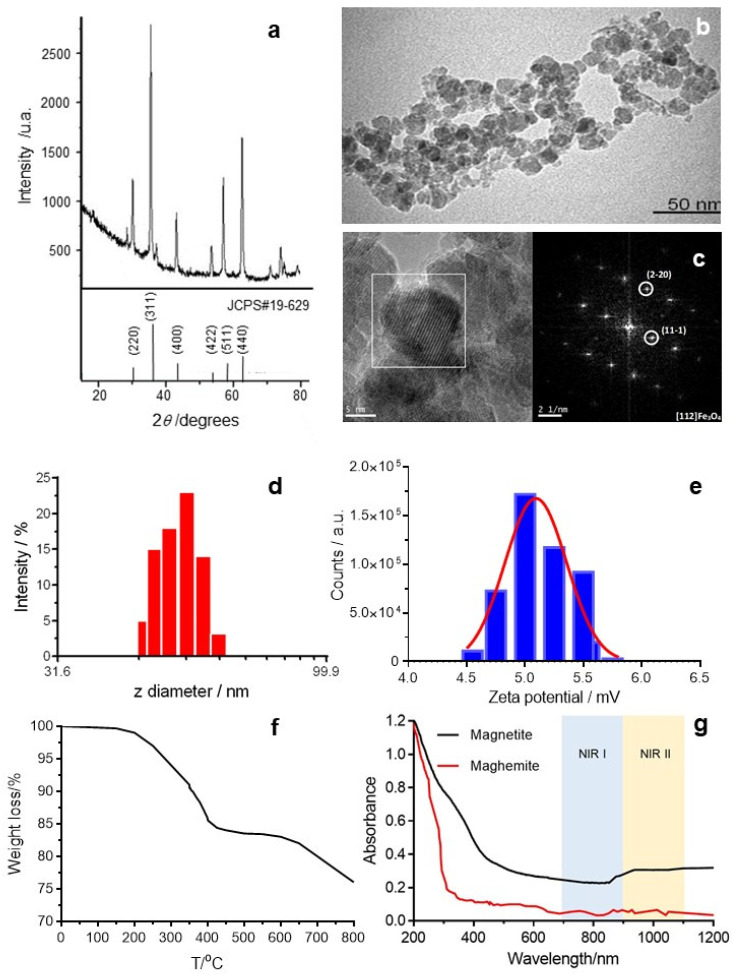
(**a**) X-ray diffraction (XRD) pattern, (**b**) transmission electron microscopy (TEM) image, (**c**) high-resolution (HR)-TEM images and the corresponding fast Fourier transform (FFT), (**d**) hydrodynamic radius of the as-prepared ferrofluid, (**e**) ζ-potential, (**f**) thermogravimetric analysis (TGA) and (**g**) UV-Vis-near-infrared (NIR) absorption spectrum of a suspension of 4.5 mM iron oxide nanoparticles (IONs) in water and after oxidation (maghemite at the same concentration).

Figure 1a shows the XRD measurement of the as-obtained ferrofluid. The observed relatively strong peaks at 2θ values of 29.98°, 35.44°, 42.94°, 53.51°, 56.82° and 62.46° were ascribed to the (111), (220), (311), (400), (511) and (440) planes of magnetite, which match well with the database of magnetite in the JCPDS (JCPDS card number 19-629).

The mean crystal size (D_hkl_) is the crystallite size, the smaller monocrystall in the system. It is calculated by the Scherrer equation which relates the size to the broadening of a peak in the diffraction pattern. The Scherrer equation is given by:

(1) $$D_{\mathrm{hkl}}=\frac{0.9\lambda }{\beta \cos\theta }$$

where λ is the X-ray wavelength (0.154056 nm), 0.9 is a dimensionless shape factor, β is the half-width of the most intense XRD peak, that is the peak at 2θ = 35.44° (0.01183 rad), and θ is the half-diffraction angle or Bragg angle (17.72°). The mean crystal size was found to be 12.3 nm (D_311_). The interplanar (d_hkl_) is the spacing between planes in a family with the Miller indexes h, k and l. At the plane (311) it can be obtained from:

(2) $$d_{311}=\frac{\lambda }{2\;\sin\theta }=0.2531\;\mathrm{nm}$$

The lattice constant (a) for a cubic structure calculated from:

(3) $$d_{\mathrm{hkl}}=\frac{a}{\sqrt{h^{2}+k^{2}+l^{2}}}$$

was 0.8394 nm.

Figure 1b,c shows the transmission electron microscopy (TEM) and high-resolution TEM (HRTEM) images, respectively, of the ferrofluid. The TEM image illustrates aggregated IONs while the HRTEM image shows their high crystallization behavior. The observed aggregation was due to the preparation of samples for TEM. In suspension, these nanoparticles are very stable. The primary particles were estimated as 13.3 nm in diameter with almost spherical shapes. Figure 1d shows the size distribution in intensity of the IONs obtained by DLS. The z-average diameter was 54 ± 13 nm (hydrodynamic diameter) and the polydispersity index (PI) was 0.10 ± 0.06. The nanoparticles presented a low surface charge at pH 6.5 (ζ-potential ∼5 mV) (Figure 1e) due to the hydroxyl groups of the PEG adsorbed onto the iron nanoparticle. A TGA curve performed over a temperature range of 160–450 °C gave a weight loss of 14.5% (Figure 1f). The loss of weight was a consequence of desorption and subsequent decomposition of PEG. The content in iron determined by inductively coupled plasma-optical emission spectrometry (ICP-OES) was 595 µg of Fe by g of ferrofluid.

The excellent photothermal performance of IONs in the second biological window is due to the intervalence charge transfer (IVCT) transition that such nanoparticles possess. The charge-transfer transitions between Fe^2+^ and Fe^3+^ ions in magnetite provide an absorption band in the NIR region (Figure 1g). It is worth noting that this transition is not present in maghemite [21]. The absorption spectrum of IONs shows a dip in the 700–800 nm region, which covers the largest part of the first biological widow, but it increases considerably in the second biological window range [34,35].

**Figure 2:**
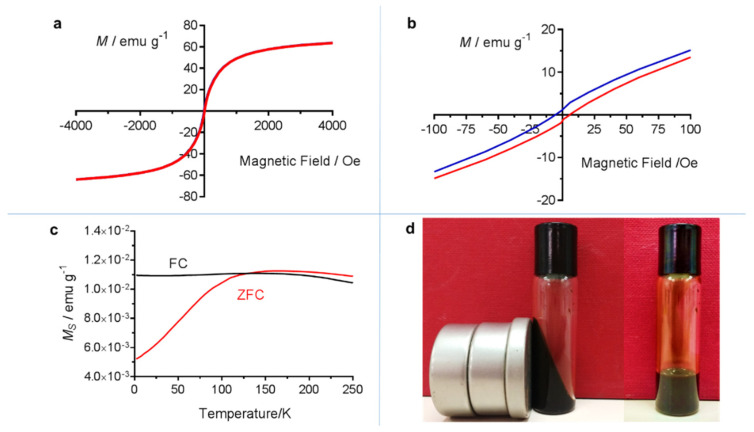
(**a**) Magnetization plot at 300 K of the as-prepared fluid; (**b**) Enlargement of the hysteresis loop of Figure 2a in the low field region; (**c**) Temperature-dependent ZFC-FC magnetization curves of the as-prepared ferrofluid measured at 50 Oe; (**d**) Effect of an external magnet on the ferrofluid.

Figure 2a presents the magnetization–magnetic field (M–H) plots at 300 K of IONs. They show a sharp slope and a lack of a hysteresis loop, pointing out superparamagnetic behavior. The saturation magnetization (Ms) was estimated to be 65 emu g^−1^ at 5 kOe, lower than of bulk Fe_3_O_4_ (77 emu g^−1^) but concordant with the percentage of polymer coating the magnetite. According to the magnetization, we obtained a polymer coating of 15.6%, similar to the 14.5% obtained by TGA. A magnetic squareness ratio (ratio between the remanent magnetization (Mr = 0.98 emu g^−1^) and the saturation magnetization) of 0.015 was obtained, indicating that these IONs are superparamagnetic at room temperature since any value < 0.5 confirms the existence of a single domain. Moreover, a coercivity of 7 Oe was observed (Figure 2b). This low value of coercivity indicates a low resistance of such material to becoming demagnetized. Concerning the temperature-dependent magnetization. Concerning zero-field-cooled-field-cooled (ZFC–FC) curves between 5 and 300 K at 50 Oe, the collected data of IONs are reported in Figure 2c. To create a ZFC curve, the sample was cooled to 5 K in the absence of a magnetic field. Then, a weak field (50 Oe) was applied and the sample slowly warmed. When the afforded thermal energy reached a value, individual spins started to align with the applied field. The alignment reached a maximum at 130 K, the blocking temperature (*T*_B_). After that temperature, the alignment decreased as a function of the inverse of the temperature as thermal energy exceeds the energy obtained by the alignment of the spins. *T*_B_ is well below room temperature pointing out that the synthesized IONs are superparamagnetic at physiological temperature as required for in vivo applications. The FC curve was produced at this point by re-cooling the sample in the presence of the same field. There was no hysteresis above *T*_B_ where the sample is superparamagnetic. At *T*_B_ the spins become blocked and unable to reorient themselves, and they keep in their maximally aligned state [37]. At a temperature lower than *T*_B_, the curves FC and ZFC separate each other, pointing out that the material shows ferromagnetic behavior. Figure 2d shows the response of the ferrofluid to an external magnetic field.

### 2.2. Photothermal Properties of IONs

**Figure 3:**
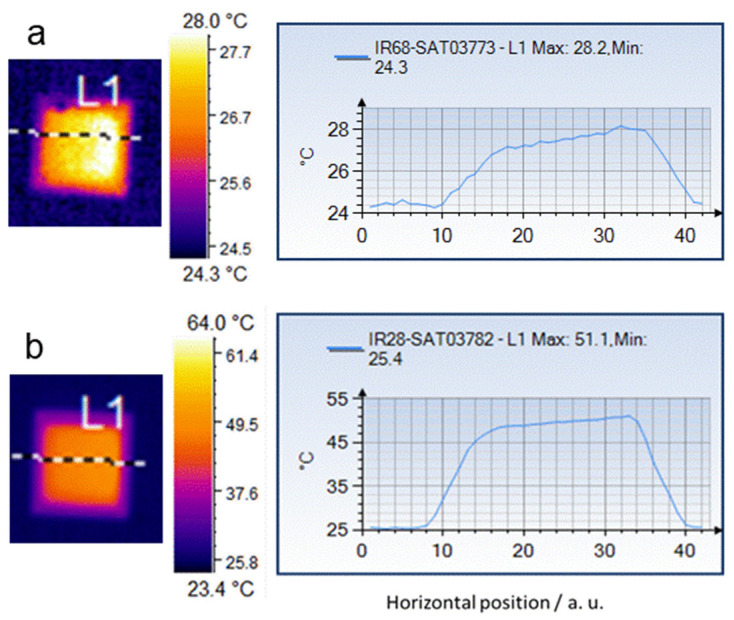
Thermal image acquired by the IR camera on (**a**) water and (**b**) the sample at 51 mg mL^−1^ concentration at the intermediate power (8.7 W cm^−2^) after 8 min of irradiation. The temperature measured at the cross-sectional L1 line (left) is recorded in the picture on the right. In this case, a maximal value of 28.2 °C and 51.1 °C was obtained for water and the sample, respectively. The laser beam went from right to left through the center of the wall of the cuvette.

Photothermal effects of IONs were first tested in aqueous suspensions at three iron concentrations (51, 127.5 and 255 mg L^−1^) at three powers (3.5, 8.7 and 14 W cm^−2^). We used a total power laser relatively low (from 0.3 W to 1.3 W) but these powers are applied on a small beam laser diameter (3.6 mm) and, for this reason, the resulting power densities are high. The 1064 nm laser beam was directed through the quartz cuvette with the different suspensions, and temperature measurements were obtained with the IR camera. The incident (as well as the transmitted) laser power was measured with a calibrated thermopile (Laserpoint model AHA-05-D20-BBF). The temperature increase was recorded along the sample for 10 min. As can be seen in Figure 3, after a few minutes of irradiation there were small temperature differences along the measured area. The maximum recorded value in the area was chosen as the characteristic temperature for each time.

**Figure 4:**
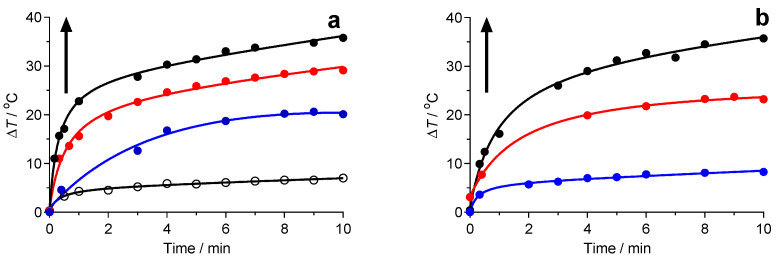
Plots of the temperature increase versus time during laser irradiation Concentration-dependent thermogenesis of IONs at (**a**) various concentrations at a power density of 8.7 W cm^−2^ (black dots: 255 mg L^−1^; red dots: 127.5 mg L^−1^; blue dots: 51 mg L^−1^; open dots: Water), and (**b**) at various laser power densities at an iron concentration of 51 mg L^−1^ (black dots: 14 W cm^−2^; red dots: 8.7 W cm^−2^; blue dots: 3.5 W cm^−2^).

The photothermal effect of IONs in water upon laser irradiation was investigated at several iron concentrations and power densities of the 1064 nm laser. At any power density, an evident concentration-dependent temperature increase was observed (Figure 4a), whereas the pure water (double deionized water) showed slight changes in temperature, especially for the lowest power density (1.5 °C at 3.5 W cm^−2^). It is important to remark that in the second spectral window, optical absorption does not disappear entirely (averaged water absorption coefficient is close to 0.5 cm^−1^) [13]. Concerning the samples, a temperature increase of 20.5 °C was detected at the lowest concentration (51 mg L^−1^) within 10 min, whereas at the high concentration (255 mg L^−1^) an increase of 35 °C was recorded. This value was extremely significant in comparison with that obtained with pure water (~6.7 °C). In the same manner, the temperature change of the IONs at a constant concentration (51 mg L^−1^) also showed laser power-dependent behavior (Figure 4b). The temperature of IONs increased up to 63.5 °C at a power density of 14 W cm^−2^, 51.5 °C at a power density of 8.7 W cm^−2^ and ~36 °C at 3.5 W cm^−2^ within 10 min of laser irradiation. The temperatures reached after this time for all of the experiments carried out can be found in Table S1 of the Supplementary Materials. As expected, heating increased when exposing the samples at high power densities, and it also increased with the nanoparticle concentration. Available preclinical photothermal studies have used power densities up to 3–4 W/cm^2^ [38,39]. For biomedical applications, irradiation with high power density can be used at short exposure times to ablate tumoral zones or to obtain a rapid increase of temperature.

**Figure 5:**
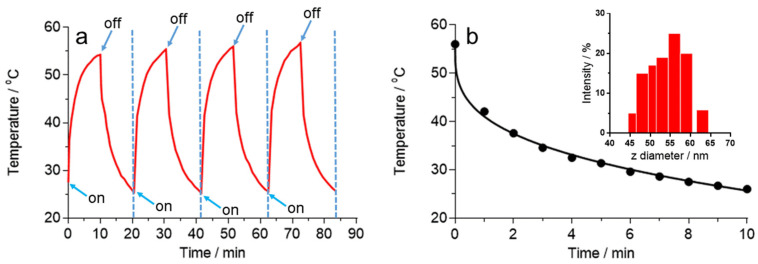
Photothermal stability of IONs. (**a**) Plot of temperature change of the IONs in water over four on/off cycles of 1064 nm laser irradiation; the temperature increased from an ambient value of 25 °C to an equilibrium value of 56 °C during continuous irradiation for 600 s. (**b**) Plot of temperature decay during the cooling period. Inset: Size distribution of IONs after being submitted to the four irradiation cycles.

To explore the photothermal stability of IONs, multiple cycles of laser irradiation were performed with an iron concentration of 127.5 mg L^−1^ at a power density of 8.7 W cm^−2^. After four continuous heating/cooling cycles, the changes in temperature were consistent (Figure 5a). From this plot, we can observe that IONs exhibit good thermogenic stability during the whole process. The temperature increased by around 30 °C when the suspension was irradiated for 600 s and this increase did not vary significantly with further irradiation. The maximal temperature was achieved when an equilibrium between the heat input and output was reached. Contrary to the ferrofluid, the temperature of the control water increased by only 6.7 °C. Besides this photothermal property, IONs present great stability during photothermal heating at temperatures above 770 K, displaying no change in size or crystallinity [24]. 

Using the heating profile of pure water, the *Q*_dis_ was measured to be 0.319 W. The cooling cycle obtained in the study of the photothermal stability of IONs was used to determine the rate of heat transfer from the IONs to the environment. Figure 5b displays the temperature decay when IONs are under the cooling period after the irradiation of the sample for 10 min. Mathematically, the temperature follows an exponential decay equation that is deduced from the energy balance when there is no heat input:

(4) $$T=T_{amb}+\left(T_{max}-T_{amb}\right)e^{-\frac{t}{\tau_{s}}}$$

where *t* = 0 is assigned when the temperature is *T_max;_* *T_amb_* is the room temperature. By fitting the values of the temperature decays shown in Figure 5a, the time constant *τs* of 220 s was determined. 

Photothermal conversion efficiency (*η*), determined according to Equation (5), is an essential parameter for the assessment of any photothermal agent. The heat transfer factor *hS* was evaluated and a value of 25 ± 1 mW K^−1^ was obtained.

The calculated *η* values were 76%, 70% and 80% for 255 mg L^−1^, 127.5 mg L^−1^ and 51 mg L^−1^ of iron, respectively (average *η* = 75.3 ± 5.0) (Figure S1). These values demonstrate that IONs present a good photothermal performance in the second biological window. It is known that IONs possess low molar absorption coefficient in the first biological window (15 M^−1^ cm^−1^ at 808 nm) and, in consequence, their photothermal performance is poor. In contrast, we have determined a molar absorption coefficient of 182 M^−1^ cm^−1^ at 1064 nm (Figure S1). Moreover, the photothermal effectiveness of IONs is dependent upon particle size, since the molar absorption coefficient increases with material size. In this way, Shen et al. have reported that clustered IONs were more efficient that the individual IONs in increasing the temperature at 808 nm [26]. The IONs used here (13.3 nm) can be considered as superparamagnetic particles of high size. Johnson et al. have proved by experiments and simulation that the heating ability is related to the volume of the nanoparticles, rather than the absorptivity of the particles. This dependence is associated to the fact that particles with larger heat capacities can heat larger volumes of their surrounding media for larger periods [25]. 

## 3. Materials and Methods 

### 3.1. Materials 

Ferric chloride hexahydrate (FeCl_3_·6H_2_O) and ferrous chloride tetrahydrate (FeCl_2_·4H_2_O) were purchased from Sigma-Aldrich (St. Louis, MO, USA). Polyethylene glycol (PEG) (Mn = 4000 g mol^−1^) was obtained from VWR International (Barcelona, Spain). Ammonium hydroxide (NH_4_OH, 25%) was purchased from Panreac (Barcelona, Spain). Deionized Millipore Milli-Q water was used in all experiments. A strong neodymium-iron-boron (Nd_2_Fe_12_B) magnet (1.2 T) was obtained from Halde GAC (Barcelona, Spain).

### 3.2. Synthesis of IONs 

IONS coated with PEG were prepared by coprecipitation of iron salts according to the reported method [40]. In this case, 3 g of PEG was added to a volume of 5 mL of water. After dissolving the polymer, 0.435 g of FeCl_3_·6H_2_O and 0.16 g of FeCl_2_·4H_2_O (2:1 molar ratio of FeCl_3_/FeCl_2_) were added. When the iron salts and PEG were well dissolved, a solution (10 mL) of a 0.75 M NH_4_OH was added under intense mechanical stirring at a speed of 0.6 mL min^−1^. The resulting ferrofluid was washed, collected with an external magnetic field and sonicated. Maghemite was obtained by boiling IONs with a solution of 0.8 mol of ferric nitrate for 30 min.

### 3.3. Characterization of IONs

The crystalline phase of the coated nanoparticles was identified by X-ray diffraction (XRD) using a Bragg-Brentano *θ*/2*θ* Siemens D-500 diffractometer (radius = 215.5 mm) equipped with a Cu Kα radiation source. The morphology of nanoparticles was observed by transmission electron microscopy (TEM) with a Jeol 1010 microscope and an HR-TEM Jeol 2010F microscope (Jeol, Japan) operating at an accelerating voltage of 80 kV and 200 kV, respectively. Images were recorded with a Megaview III camera, and the acquisition was accomplished with Soft-Imaging software (SIS, Germany). The hydrodynamic diameter of nanoparticles was determined by dynamic light scattering (DLS) at a fixed scattering angle of 90° with a Zetasizer Nano (Malvern, United Kingdom) at 25 °C. To perform this measurement, nanoparticles from the stock solution were dispersed in distillated water until 0.1 g L^−1^ solid content. Particle size distribution was determined by the polydispersity index (PI); this value ranges from 0.0 for an entirely monodisperse sample to 1.0 for a polydisperse sample. Absorption spectrum was obtained across the UV/Vis/NIR range (Perkin Elmer Lambda 950, Waltham, MA, USA). The iron concentration of the nanoparticles was determined with an inductively coupled plasma optical emission spectroscope (ICP-OES, Perkin Elmer model Optima 3200RL, USA). Isothermal magnetization was determined in a superconducting quantum interference device (SQUID) magnetometer (Quantum design MPMS XL) at 300 K. For this determination, a few milligrams of the sample were lyophilized and the external magnetic field was swept from +5000 to –5000 Oe, and then back to +5000 Oe. The saturation magnetization values were normalized to the mass of nanoparticles to yield the specific magnetization, *M*_s_ (emu g^−1^). Temperature-dependent curves were obtained after first cooling samples from 300 K in zero field applying 50 Oe (zero-field cooled [ZFC] curve), and then performing measurements upon warming (field-cooled [FC] curve). The content of PEG coating the iron oxide nanoparticles was determined by thermogravimetric analysis (TGA) using a TGA/SDTA851e (Mettler Toledo) with a 10 °C/min heating rate under nitrogen atmosphere (50 mL min^−1^). The measurement was taken from room temperature up to 800 °C. The ζ-potential of nanoparticles was measured by Doppler microelectrophoresis using a Zeta Sizer Nano ZS (Malvern, United Kingdom). 

### 3.4. Photothermal Properties of IONs

IONs with different iron concentrations (51, 127.5 and 255 mg L^−1^, equivalent to 0.91, 2.25 and 4.5 mM) dispersed in water (3.0 mL) were poured in quartz cuvettes (total volume of 4.0 mL) and irradiated by a collimated beam (spot size 10.2 mm^2^) of a continuous-wave Nd:YAG laser with a center wavelength of 1064 nm for 10 min (Basel Lasertech LBI6000). The temperature of the sample was recorded with an IR thermal camera G90 (Satir, Dogheda, Ireland). Water was used as the control. To evaluate the influence on the power density on the increase of temperature, the same ION suspensions were irradiated for 10 min with the different power densities of 3.5, 8.7 and 14 W/cm^2^, respectively. The photothermal stability of IONs was also investigated. An ION suspension with iron concentration of 127.5 mg L^−1^ was irradiated with the NIR laser for 10 min, followed by turning off the laser for cooling the sample to room temperature. This cycle was repeated four times. To determine the photothermal conversion efficiency (*η*) of IONs, a suspension of 127.5 mg L^−1^ was continuously irradiated with the above laser until reaching a steady temperature, and then the laser was turned off. After that, the suspension was allowed to naturally cool to room temperature. 

The photothermal conversion efficiency was determined using the following equation:

(5) $$\eta =\frac{hS\left(T_{\max}-T_{amb}\right)-Q_{dis}}{I\left(1-10^{-A}\right)}$$

where *h* is the heat transfer coefficient, *S* is the surface area of the heat transfer to the surroundings, *T*_max_ is the maximum equilibrium temperature, *T_amb_* is the ambient temperature of the surroundings, *I* is the laser power, *A* is the absorbance of PA at the emission wavelength of the laser, that is 1064 nm, and *Q_dis_* is the heat dissipated from the light absorbed by the solvent and the container. It was measured using a quartz cuvette cell containing only double deionized water. The term *hS* is determined by fitting the rate of temperature change when the laser is on to:

(6) $$T=T_{amb}+\frac{Q_{i}}{hS}\left(1-e^{-\frac{t}{\tau_{s}}}\right)$$

where *Q_i_* is the laser power absorbed in the sample, *t* is the time at which the temperature *T* is reached, and *τs* is the time constant obtained from the cooling period after irradiating the sample [41].

## 4. Conclusions

We prepared PEG-coated magnetite nanoparticles (IONs) by an aqueous method under ambient conditions. Such non-functionalized IONs can be used as PAs in the second biological window themselves since they are able to absorb NIR radiation at 1064 nm at iron concentrations from 51 to 255 mg L^−1^ and transform it into heat, assessing the excellent photothermal conversion behavior of IONs. Moreover, the scattering that nanoparticles provoke at the second biological window is lower than in the shorter wavelengths. Hence, the efficiency of IONs in PTT is demonstrated, concretely in the second biological window. To the great potential of IONs to be used as PA, the nanoparticles add their low toxicity, as well as their easy degradation in vivo, where iron homeostasis is assumed by the iron-storage protein, ferritin. In conclusion, IONs are promising photothermal agents in the second biological window.

## Supplementary Materials

The following are available online. Figure S1: Plot of the photothermal conversion efficiency with respect to iron molar concentration, Figure S2: Plot of the absorbance at 1064 nm with respect to iron molar concentration, and Table S1: Values of temperature reached after 10 min of irradiation at three power densities and three concentrations.
