# Supplementary material for: Superparamagnetic Nanoparticles with Efficient Near-Infrared Photothermal Effect at the Second Biological Window
Source: Molecules. 2020 Nov 14;25(22):5315. doi: 10.3390/molecules25225315 (PMC7696853; doi:10.3390/molecules25225315)
Supplement: Supplementary file 1 [file molecules-25-05315-s001.pdf]

Supplementary Material

Superparamagnetic nanoparticles with efficient near-infrared photothermal effect at the second biological window

Maria Antònia Busquets[1,3], Juan Marcos Fernández[2,3], Pedro Serra[2,3], Joan Estelrich[1,3,*]

[1] Department of Pharmacy, Pharmaceutical Technology and Physical Chemistry, Universitat de Barcelona Avda Joan XXIII, 27-31. 08028 Barcelona (Catalonia, Spain)
[2] Department of Applied Physics, Universitat de Barcelona. Martí i Franquès 1. 08028 Barcelona (Catalonia, Spain)
[3] Institut de Nanociència i Nanotecnologia (IN[2]UB), Universitat de Barcelona,
* Correspondence: joanestelrich@ub.edu.Tel.: + 34934024559 (JE)

Power density /W cm^{-2}	IONs $_{concentration}$ / mg L^{-1}	Temperature after 10 min /^{o}C
3.5	0	28.7
	51	35.9
	127.5	37.6
	255	40.1
8.7	0	33.7
	51	46.4
	127.5	51.1
	255	62.2
14	0	35.8
	51	63.5
	127.5	68.2
	255	75.1

Table S1. Values of temperature after 10 min of irradiation at three power densities and at three concentrations. An ION concentration of 0 mg L^{-1} represents a sample containing solely water. The initial temperature (time = 0) was ranging from 27.2 to 28.6 ^{o}C.

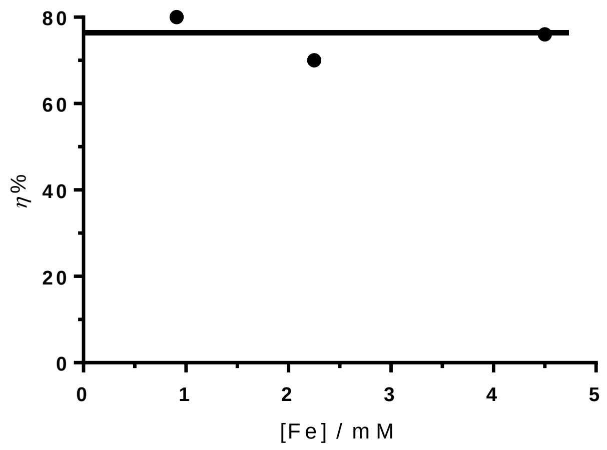

Figure S1. Photothermal conversion efficiency with respect to iron molar concentration.

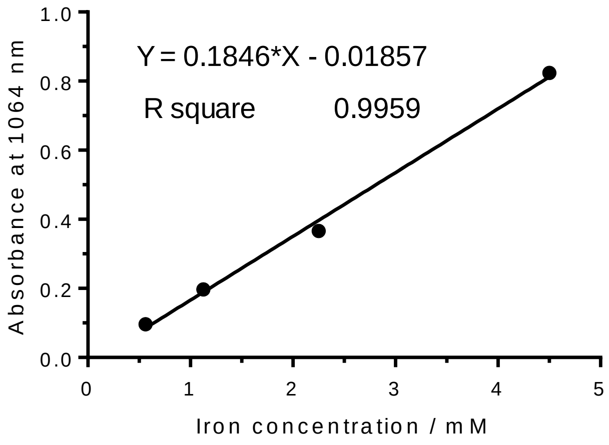

Figure S2. Linear regression corresponding to the changes in absorbance at 1064 nm with increasing iron concentration.
